# Unraveling the Mystery of Desmoid Tumors: Insights From a Moroccan Tertiary Center

**DOI:** 10.7759/cureus.57768

**Published:** 2024-04-07

**Authors:** Salma Najem, Soukaina Bekkouche, Amine Benslimane, Sarah Naciri, Hanane Inrhaouen, Ibrahim EL Ghissassi, Saber Boutayeb, Hind Mrabti, Hassan Errihani

**Affiliations:** 1 Department of Medical Oncology, National Institute of Oncology, Faculty of Medicine, Mohammed V Faculty, Rabat, MAR

**Keywords:** surgery, y secretase inhibitor, chemotherapy, active surveillance, desmoid tumors

## Abstract

Desmoid tumors (DTs) are rare, aggressive malignancies developing from clonal fibroblastic proliferation originating from soft tissues. Despite their low metastatic potential, their invasiveness towards neighboring organs and a high recurrence rate contribute significantly to morbidity and mortality, thereby impacting the quality of life of patients. Several therapeutic options are available, but standardized protocols are lacking. In this study, we reviewed 14 cases of DT retrospectively over a period of 15 years, from September 2008 to December 2023. The most prevalent tumor locations were in the extremities, and the majority of patients were female. We identified risk factors in two patients, those being surgical trauma and familial adenomatous polyposis (FAP). Half of the patients underwent surgery for DT, and two received salvage radiotherapy. Systemic therapy was used in the first and second lines and comprised of chemotherapy, endocrine therapy, and non-steroidal anti-inflammatory drugs (NSAI).

Active surveillance was proposed in three patients. This is the first retrospective study to assess the characteristics of DT in Moroccan patients in a tertiary care setting. It aims to shed light on the challenges faced in treating these rare tumors in the context of a lack of therapeutic standardization.

## Introduction

Desmoid tumor (DT) is a rare, histologically benign fibromatosis without a metastatic potential. Still, a high local invasiveness and recurrence rate [1], lead to compression of neighboring organs and vital structures and resulting in pain, and impairment of nerves and organ function, Thereby, leading to a degradation of the quality of life of patients [1]. Two main molecular anomalies were identified as being responsible for DT the mutation in the catenin gene named cadherin-associated protein (CTNNB1) responsible for the sporadic form and the germline mutation of the adenomatous polyposis coligene (APC) [1].

Diagnosing DT is difficult due to its varied morphological characteristics and unpredictable clinical manifestations, and its treatment can be challenging, especially when the tumor is located at an atypical site [2-4]. However, there has been a paradigm shift in favor of a conservative strategy with active surveillance as an initial step [3,5]. Furthermore, interest in this entity was rekindled this year owing to a new molecule, Nirogacestat, which acts on the Notch pathway normally overactivated in those tumors. This molecule proved its efficacy in the DEFI trial over a placebo in progressive DT in need of a systemic therapy, leading for the first time to the approval of a drug by the FDA in 2023 in this indication [3].

In Morocco, DT treatment is not standardized for a variety of reasons, the most important of which is a lack of medical and surgical expertise in rare tumors; however, this is changing as multidisciplinary consultation meetings are established. As a result, we developed this retrospective series to highlight the characteristics, clinical trajectory, and therapeutic options used in Moroccan patients with DT, allowing us to demonstrate our experience as a cancer management reference center.

## Materials and methods

 Study design

A retrospective investigation encompassing all cases of DT was conducted in a tertiary cancer center (The National Institute of Oncology of Rabat, Morocco). The study was approved by the local institutional review board, and the database was retrospectively reviewed for all patients diagnosed with DT from September 2008 to December 2023.

The diagnosis relied on clinical, radiological, and histological criteria. Patients with DT who were histologically confirmed, treated at the study location, and had complete medical records were the only ones included. Clinical criteria most likely comprised symptoms reported by patients as well as signs seen during physical examinations. Tumor location, size, and characteristics have been ascertained by radiological investigations using imaging techniques such as computed tomography (CT) and Magnetic resonance imaging (MRI) scans. Before any treatment, including surgery, chemotherapy, or surveillance, an initial assessment was made, repeated every three months during treatment to evaluate response, and every six months after treatment cessation. The tumor change was evaluated with response evaluation criteria in solid tumors (RECIST) guidelines (version 1.1).

Data collection

Information gathered from the medical files for this retrospective analysis includes age at diagnosis, gender, diagnosis date, location of the primary tumor, size, type of treatment received (surgery, radiotherapy, systemic treatment), surgical margins, recurrence occurrences, progression dates, last follow-up date, and survival status.

Lesions located at the thigh, arm, and shoulder were categorized as extremity lesions. Due to their limited number in our study (n = 1), neck lesions were grouped with extremity lesions. Surgery with margins was categorized as gross positive (R2), microscopic positive (R1), or negative (R0). Radiotherapy as well as systemic therapies, and their duration were recorded. Recurrence was defined as either histology-proven or radiologic evidence of disease.

Statistical analysis

To conduct statistical analysis, progression-free survival (PFS) was employed and calculated from the date of diagnosis until the event occurrence or last follow-up. The event was defined as either a disease-related death or a disease recurrence.

We evaluated survival rates using the Kaplan-Meier technique. SPSS software (SPSS for Windows, version 24.0, IBM Corp., Armonk, NY, USA) was used to conduct statistical analysis. Disease-free survival and overall survival were calculated using the Kaplan-Meier method.

## Results

The study enrolled 14 patients with a median age of 34.5 years (ranging from 19 to 73 years). Most of them were female, nine out of 14 (64.2%), and only five patients were male (35.8%) (Table 1).

**Table 1:** Main patients characteristics

Patient no	Age of diagnosis	Gender	Disease area	Size (cm)
1	34	Female	Intraabdominal	13,2
2	30	Female	thigh	7,5
3	45	Female	Thigh	18,1
4	17	Female	Thigh	19,2
5	19	Female	Abdominal wall	6,1
6	31	Female	Iliac wing	13,6
7	19	Male	Intraabdominal	15,5
8	39	Female	Chest wall	7
9	67	Male	thigh	3,1
10	35	Female	Shoulder + arm	8
11	69	Female	Intra-abdominal	8
12	69	Male	Hip	6,5
13	40	Male	Shoulder	5,8
14	24	Male	Neck	10,2

One patient had a surgical procedure at the tumor site for fibrous fasciitis seven months prior to the appearance of the DT. Another patient had a history of locally diagnosed breast cancer with conservative surgery followed by adjuvant chemotherapy, radiotherapy, and five years of aromatase-inhibitor therapy. One patient with Gardner syndrome underwent a preventive total colectomy for familial adenomatous polyposis (FAP) (Table 2).

**Table 2:** Characteristics of initial treatments

Patient no	Surgery	Radiotherapy	Systemic therapy	Recurrence or progression	Current status
1	No	No	Vinorelbine (30 months)	No	Alive
2	No	No	Tamoxifen+AINS ( 10 months)	No	Alive
3	R0	No	No	Yes	Alive
4	No	No	Vinorelbine (11 months)	No	Alive
5	R0	No	No	Yes	Alive
6	R1 (50 Gy, 2Gy per fraction)	Yes	No	No	Alive
7	No	No	Methotrexate-Vinblastine ( 24 months)	No	Alive
8	R2 (56 Gy, 2Gy per fraction)	Yes	No	Yes	Alive
9	No	No	Vinorelbine (2 months)	Yes	Dead
10	R0	Yes	Tamoxifen	Yes	Alive
11	R0	No	No	No	Alive
12	No	No	No	Yes	Alive
13	No	No	No	No	Alive
14	R0	No	Navelbine (12months)	Yes	Alive

The most common symptom reported was a palpable mass (n = 12), with three patients experiencing severe pain. Tumor sites were variable: nine in the extremities (63.3%), three (21.5%) in the abdominal region, one in the abdominal wall (7.1%), and one in the thoracic wall (7.1%). While 13 patients (92.9%) had a solitary lesion, only one patient (7.1%) had two lesions. The median size of the tumor was 8 cm (range, 3.1-19.2 cm). Out of the 14 patients, only one exhibited two locations in the upper extremity (shoulder plus right arm) (Table 1).

The histological diagnosis was made by a core-needle biopsy for all patients. Seven patients (50%) underwent wide surgical resections. Among them, five patients achieved wide (R0) resection, one had marginal (R1) resection, and one patient underwent intralesional (R2) resection (Table 2). Additionally, the two patients who had undergone R1 and R2 resections received adjuvant radiation therapy with a median dose of 53 Gy. While no patient had received radiotherapy as the primary treatment (Table 2), seven patients (50%) received systemic therapy, with five undergoing primary systemic treatment. Of these, four were administered chemotherapy for an average of 16 months (ranging from two to 30 months), with three of them having vinorelbine and one receiving methotrexate associated with vinblastine, while one received tamoxifen for 10 months. The two remaining patients received systemic treatment at progression, one with vinorelbine and the other with tamoxifen and non-steroid anti-inflammatory drugs (AINS) mainly celecoxib for a duration of 12 and 10 months, respectively. Three patients (21.5%) underwent active surveillance, two initially and one following disease progression.

Local recurrence (LR) was observed in seven (50%) patients, with a median time interval of 42 months (ranging from two to 180 months). Among the seven patients experiencing LR, one had positive margins following initial surgery (Table 2). Two patients underwent subsequent resection, three were put under observation, one patient had systemic therapy with vinorelbine, and one patient passed away under initial systemic therapy. Among the two patients who underwent repeat resection, one experienced a second relapse, treated with radiation, and a third relapse was put under tamoxifen (Table 3).

**Table 3:** Secondary treatments in patients with recurrence or progression

Patient No	First line treatment	Second line treatment	Third line treatment
5	Surgery	-	-
10	Surgery	Radiotherapy	Tamoxifen

## Discussion

DT are rare and represent 0.03% of all neoplasms [1], with an incidence of five to six cases per one million population per year [4,6]. Unfortunately, in Morocco, we do not have an exact estimate of the incidence of these tumors compared to more common ones, but our series seems to confirm the rarity of this pathology, since between 2008 and 2022, only 14 cases were recorded in our tertiary center, which is regarded as Morocco's reference center for cancer treatment. This seems to be in line with many retrospective series published throughout the world, except for a Polish study that can be considered one of the largest retrospective cohorts with 363 patients [7].

This tumor tends to occur in young and old patients, with a peak age of 30-40 years and a female predominance [8]. This has led to considering women of childbearing age as a group at risk of developing these rare tumors, but it remains a subject of controversy and debate [8,9]. Recently, a French study tried to answer this question by estimating the risk of events in selected young female patients from a prospective cohort of DT according to the use of hormonal contraception and pregnancy status. The results were quite interesting, as they showed an increased risk of events 24 months after pregnancy, whereas hormonal contraception did not show a significant association [9]. Without exception to the rule, our series included more women than men, 88% of whom were of childbearing age, and half of these young women had children, but the time between pregnancy and disease onset was not recorded in their medical records.

Other risk factors reported in the literature to be associated with DT were found in our patients, such as trauma, especially surgical trauma, which was present in two patients (14.2% of all our patients), and one of these patients had FAP as part of Gardner's syndrome [10]. The prevalence of DT in FAP is 10%-25%, and more than 70% of cases are noted after abdominal surgery, with the majority of cases occurring within five years of abdominal surgery, as was the case in our patient, who presented with a DT two years after a prophylactic colectomy for his Gardner syndrome [8]. It is important to note that, to date, there is no evidence linking the type of surgery with the occurrence of DT [11].

These FAP-associated DT have been shown to harbor an APC germline mutation, Moreover, 10%-15% of sporadic DT cases may be caused by these mutations [1,8]. The loss of the APC gene allows this cytosolic protein to move into the nuclei and increases the cellular component of the cell cycle [1]. On the other hand, the mutation responsible for most cases of sporadic DT is the β-catenin mutation oncogene in the N-terminal region of CTNNB1 (80%-95%), which leads to the cytoplasmic accumulation of β-catenin and its subsequent translocation to the nucleus, resulting in dysregulation of the Wnt/APC/B-catenin pathway [6].

The Desmoid Tumor (DT) Working Group released a consensus in 2019 that suggested having a mutational analysis done and having the diagnosis of DT confirmed by a skilled soft tissue pathologist. Since APC and CTNNB1 mutations are mutually exclusive, the detection of a somatic CTNNB1 mutation helps to exclude a syndromic condition [6]. In our center, somatic mutations of CTNNB1 are systematically analyzed to confirm the diagnosis, and we found it in only half of the patients, but this can be explained by the fact that certain slides were not read in our center. However, the presence of this mutation is not always systematic and can be missed in almost 20% of cases, suggesting rather the presence of a mutation of the APC gene and requiring, in this situation, a colonoscopy to exclude an underlying FAP [6].

Our results in terms of location seem to concur with several series and literature reviews, with a predominance in the extremities in over 50% of patients [8], whereas other case series place abdominal locations in the first position [4,12]. We have no clear explanation for this discrepancy in the literature, but it has been suggested that age may play a role in determining tumor site [4]. For instance, Reitamo et al. discovered that extra-abdominal locations were more common in kids under the age of 15, while abdominal localization appeared to be more common in older patients [13]. Due to the small patient population, we are unable to extrapolate this deduction to our series.

Historically, surgery has long been the mainstay treatment of DT [7]. However, a high recurrence rate (up to 60%) even after surgical resection with negative margins and the results of many retrospective and prospective studies demonstrating no difference in PFS and overall survival between initial surgery and active surveillance have made it more of an option to be discussed, particularly in cases of symptomatic tumors or progressive ones [14]. Our series serves as an excellent example of this paradigm change, since the majority of patients who underwent surgery as a first line of treatment were those who were diagnosed before 2016, even though one patient had a cervical site that was thought to be difficult to resect.

It's interesting to note that three-quarters of patients with extended negative margins after surgery which is more than half of resected individuals relapsed. Contrarily, patients who did not obtain sufficient margins (microscopic invasion) experienced a wider range of outcomes, with one example showing no recurrence at all and another showing one after 15 years [14]. Since there has not been agreement on what a sufficient broad margin is, in part due to contradictory reports and findings, it seems that the objective for DT resections should be to achieve a negative surgical margin, even if it is almost microscopic [8,14,15].

Different radiotherapy techniques have been evaluated in DT, be it brachytherapy, proton therapy, or intensity-modulated radiation therapy (IMRT). These techniques have been used in postoperative and unresectable settings. Of all these techniques, proton therapy has a superior dose distribution compared to the other techniques, justifying its use in DT surrounding organs at risk. This can explain the reduction in the incidence of second malignancies too. As for brachytherapy, it has been used in combination with surgery for several years with an average dose of 45-50 Gy, providing good results in terms of local control; nevertheless, there is little evidence to support adjuvant radiation. In the unlikely event that this happens, cancer may develop 7-20 years after radiation therapy. In our center, radiation is used when surgery is unable to produce negative margins, as demonstrated by two of our patients.

But nowadays, active surveillance is the standard first approach to consider for the majority of patients [5,6,14]. Yet as simple as it can appear, this approach requires not only the availability of an MRI but also respect for close intervals, especially at the start [6]. The DT group defines active surveillance as ongoing MRI monitoring every one to two months, subsequently every three to six months [6]. The fact that the decision for active surveillance for three of our patients was made recently indicates that our center tends to follow international guidelines. Furthermore, these patients are currently under satisfactory control.

In terms of medical treatments, the example of our patients seems to translate the variety of options proposed for DT; these different options reflect a biology whose mechanisms are diverse, and which remains mysterious to this day. The medical options recommended were endocrine therapy, primarily tamoxifen, non-steroidal and steroidal anti-inflammatory agents, cytotoxic chemotherapeutic agents, and tyrosine kinase inhibitors (TKIs), but none of them are approved specifically for the treatment of DT [16].

The rationale behind using endocrine therapy is based on the fact that 30% of these tumors express hormone receptors [3], yet there is only one prospective phase II study assessing antihormonal therapy with NSAIDs and showing 36% PFS at two years; current clinical guidelines do not place this option as a viable one [14,17]. Consequently, NSAIDs are not currently considered for the treatment of DT [6,14].

Half of our patients received systemic therapy as the first and second line, with vinorelbine as the most commonly administered cytotoxic agent. When used, it is recommended to give for at least one year with more than 40 cycles; this ensures a long-lasting effect past the cessation of the treatment [6,14]. Another option used in one of our patients with a symptomatic intra-abdominal DT is MTX-VBL [18,19]. This protocol has been widely used in DT, with a disease control rate of 64%-100% [14]. Its efficacy has been demonstrated in prospective studies and retrospective ones with similar results in pediatric patients [6]. However, TKIs such as sunitinib, imatinib, sorafenib, and pazopanib are thought to have the ability to act quickly, which is why they are employed, particularly in urgent cases with aggressive and rapid clinical presentations [20,21]. Sorafenib (400 mg daily) was assessed in a placebo-controlled randomized phase III trial, which showed a two-year PFS of 71% compared to only 36% for the placebo cohort [21]. In a phase II trial led by the French Sarcoma Group, Pazopanib was even found to be superior to the MTX-VBL combination in terms of objective response and one-year PFS [21]. Nevertheless, Nirogacestat is the only treatment for progressive DT that the FDA has approved. This oral-specific reversible gamma-secretase inhibitor has demonstrated antitumoral activity in preclinical models of the disease with encouraging results [22]. The DEFI trial is the study that led to its approval by demonstrating its superiority to placebo in patients with progressive disease, reducing the time for response, increasing the complete response, achieving PFS, and improving quality of life with less pain and less symptom burden [3]. Other innovative therapies are currently being tested in phase II trials, like Tegavivint, an inhibitor of the Wnt/B-catenin pathway in pediatric patients [14].

Even with its small sample size, our study shows that DT are a diverse group with varying clinical courses that can be stabilized with non-invasive medical therapies or managed with a watch-and-wait approach for an extended period of time. It should be noted, however, that surgery does not ensure that a tumor will not recur. Large-scale prospective cooperative research is necessary and will contribute to the advancement of the management of these orphan tumors.

## Conclusions

According to our experience, half of our patients had a recurrence following the various treatment methods we tried, demonstrating that the difficulties encountered in the treatment of DT appear to be universal, which supports the need for extensive research to pinpoint molecular traits and prognostic variables. This will be essential for figuring out the best course of action that is specific to each scenario.
